# A *gal4* insertion in the *rx3* locus as a tool for visualization and manipulation of eye fated cells in zebrafish

**DOI:** 10.1186/s40659-025-00656-9

**Published:** 2025-11-23

**Authors:** María J. Vásquez-Ramírez, Aarón Villanueva, Esteban Lira, Daniel Nahuelpan, Koichi Kawakami, Leonardo E. Valdivia

**Affiliations:** 1 Center for Integrative Biology, Facultad de Ciencias, Universidad Mayor, Santiago, Chile; 2 Laboratory of Molecular and Developmental Biology, National Institute of Genetics, Mishima, Japan; 3 Choju Medical Institute, Fukushimura Hospital, Toyohashi, Japan; 4 Escuela de Biotecnología, Facultad de Ciencias, Universidad Mayor, Santiago, Chile

**Keywords:** Eye, Morphogenesis, Zebrafish, Genetics, G*al4/UAS*

## Abstract

**Background:**

The *rx3* gene encodes a transcription factor essential for eye development in zebrafish. Despite its importance, the spatiotemporal dynamics of gene expression during embryogenesis are not fully understood. This knowledge gap, coupled with the limitations of existing genetic tools, highlights the need for novel and more faithful reporters. In this study, we characterized a new transgenic zebrafish line with an insertion of *gal4* transcriptional activator in the second exon of the *rx3* gene, named *tg(gSAIzGFFD2459B)*, to visualize and analyse gene function.

**Results:**

In heterozygous embryos, the *gal4* insertion drives gene expression to label eye-fated progenitor cells, providing a valuable tool for dissecting the mechanisms underlying eye formation. Conversely, homozygous *gal4* insertion results in a fully penetrant eyeless phenotype, allowing for the continued tracking of *rx3*-expressing cells within the developing brain. This transgenic line permits intersectional labelling with other *UAS*-driven transgenes, enabling further exploration of gene function. Remarkably, unlike previously published *rx3* reporters that use medaka gene regulatory sequences, our line lacks ectopic expression and provides a more faithful recapitulation of endogenous *rx3* expression.

**Conclusion:**

*The tg(gSAIzGFFD2459B)* line represents a versatile platform for both studying the developmental gene regulation of eye-fated cells and enhancing our understanding of genetic determinants in vertebrate organogenesis. By reporting *rx3* transcriptional activity in both wild-type and mutant states, this transgenic tool supports the critical role of *rx3* in eye morphogenesis and offers new avenues for investigating congenital disorders of the eye and brain.

**Supplementary Information:**

The online version contains supplementary material available at 10.1186/s40659-025-00656-9.

## Introduction

Zebrafish (*Danio rerio*) is an excellent model organism for developmental biology due to its genetic similarity to humans, embryonic transparency, and rapid development. Zebrafish embryogenesis involves meticulously orchestrated processes of cell division, migration, and differentiation, resulting in detailed developmental milestones that mirror key stages of organogenesis in other vertebrates [1, 2]. This high degree of conservation makes zebrafish an invaluable model for elucidating the genetic and molecular underpinnings of developmental processes, particularly in studying genetic determinants of eye and brain formation that are often mirrored in human physiology and pathology [3, 4].

In zebrafish, eye-fated cells are specified within a single domain, known as the eye field, in the anterior neural plate [5–7]. This domain expresses a group of conserved transcription factors, with *rx3* emerging as one of the earliest and most critical markers [8, 9]. Subsequently, *rx3* plays a pivotal role in facilitating the evagination of the eye field, splitting it into two optic vesicles [10–12], which later fold into optic cups for differentiation. Extensive research further establishes a critical function for *rx3* in establishing the optic primordia and influencing neural patterning, highlighting its essential role in proper eye and brain development [8–10, 13, 14]. The importance of *rx3* as a key regulator of early embryogenesis is therefore underscored, with implications for understanding congenital eye and brain disorders [15]. To advance basic biological knowledge and uncover potential pathways implicated in developmental disorders, novel transgenic tools that enable precise, real-time visualization and manipulation of *rx3* expression in vivo are needed to provide a more comprehensive and physiologically relevant framework for studying gene function during eye formation.

To address the above-mentioned gaps, advanced transgenic techniques are pivotal. The *Gal4/UAS* system, originally adapted from yeast, has revolutionized the analysis of gene function in zebrafish by allowing precise spatial and temporal control of gene expression [16–19]. When combined with the *Tol2* transgenic approach, it becomes a robust tool to label and manipulate cells in zebrafish embryos [18, 20]. This approach facilitates the investigation of fundamental biological questions and allows for its combination with multiple *UAS*-driven transgenes, offering a multidimensional view of developmental regulation [17]. The integration of the Gal4 system into gene and enhancer traps offers distinct advantages over traditional promoter-based approaches by utilizing the native genomic context to report gene expression or enhancer activity [21].

In this study, we isolated and characterized a novel gene trap transgenic zebrafish line, *tg(gSAIzGFFD2459B)*, which represents a versatile tool for both visualizing and analysing *rx3* function. This line uniquely serves as a faithful reporter of endogenous *rx3* expression through a *gal4* insertion in the second exon, while simultaneously acting as a loss-of-function model in homozygous specimens. Our findings demonstrate that this dual capability not only reinforces the critical role of *rx3* in eye and brain development but also establishes a reliable platform for probing genetic determinants of vertebrate organogenesis and modelling related human congenital disorders.

## Methods

### Zebrafish husbandry

All animal procedures in this study were approved by the Bioethics Committee of Universidad Mayor. Adult zebrafish were maintained at the zebrafish facility of the Center for Integrative Biology, Universidad Mayor. The following fish strains were used: AB wild-type, the novel *tg(gSAIzGFFD2459B)* line (this study), and the double transgenic reporter line *tg(rx3:Gal4-VP16); tg(UAS:RFP)* [22].

Embryos were obtained by natural spawning and raised at 28.5 °C in E3 medium supplemented with methylene blue (0.00001%) in Petri dishes. Embryos were incubated under a 14-h light/10-h dark cycle and staged according to Kimmel et al. 1995 [1]. To prevent pigmentation and facilitate imaging, 1-phenyl 2-thiourea (PTU; Sigma Aldrich) was added to the embryo medium at a final concentration of 0.003%.

### Identification and isolation of the *tg(gSAIzGFFD2459B)* transgenic line

To identify transgenic lines with gene expression in developing eyes, we performed an initial screening of fluorescent images from 135 gene trap and enhancer trap lines in the zTRAP database [23]. From these lines, we selected 25 lines for adult crossing based on preliminary evidence of eye-specific expression. Embryos from these crosses were screened and imaged daily from 24 to 72 h post-fertilization (hpf) using a Leica MZ16FA dissecting microscope at the National Institute of Genetics (NIG) in Mishima, Shizuoka, Japan. We chose the *tg(gSAIzGFFD2459B)* line for further analysis due to its robust and specific transgene expression in the developing eyes, particularly within the optic primordia and retinal progenitor cells, and its discrete expression pattern in the brain.

### Genotyping PCR for gal4 insertion

Genomic DNA (gDNA) was isolated from 72 hpf *tg(gSAIzGFFD2459B)* GFP-positive larvae using the HotShot method [24]. Specimens were first lysed in 15 µL of alkaline lysis buffer (1.25 M NaOH, 0.01 M EDTA) at 95 °C for 30 min, after which 15 µL of neutralization solution (2 M Tris–HCl) was added. To confirm the *gal4* insertion, a PCR was performed using the gDNA as a template. The primer used included one primer in the transposon arm (T2_Rev: 5′ CTTCGGCTCGAGAAGTGATCT 3′) and another in the second exon of the *rx3* gene (rx3_For: 5′ GCTCCTCTCTGCTGTAGACAT 3′). The reaction yielded a 343-base pair (bp) product and the presence of which was verified on a 2% agarose gel in 1X TAE buffer. The amplicon was purified using a GFX PCR DNA and Gel Band Purification Kit (Cytiva, United Kingdom). The purified product was then sequenced using both PCR primers.

### In situ hybridization

Colorimetric in situ hybridization was performed with a specific *rx3* riboprobe, prepared as previously described [25]. The staining protocol followed the methodology of Thisse and Thisse, 2008 [26].

### Immunofluorescence and imaging

Dechorionated embryos of the *tg(gSAIzGFFD2459B; UAS:GFP)* line at various developmental stages were fixed overnight in 4% paraformaldehyde (PFA, Merck) at 4 °C. For long-term storage, embryos were washed in a graded series of PBSTr/Methanol and stored in 100% methanol at − 20 °C. For immunofluorescence staining, embryos were processed as previously described by Turner, 2014 [27]. Chicken anti-GFP (ab13970, Abcam; 1:1000) was incubated overnight at 4 °C in a blocking solution. Anti-chicken Alexa-488 (Thermo Fisher; 1:1000) was incubated with the embryos in 0.5% PBSTr (PBS supplemented with 0.5% Triton X-100, Sigma-Aldrich), with agitation at room temperature. DAPI (1:500; Invitrogen D1306) and TO-PRO-3 (1:1000; Invitrogen T3605) were used as nuclear counterstains.

Images of embryos were acquired using a Leica TCS SP8 confocal microscope and the Zeiss Lightsheet 7 fluorescence microscope, embedding the samples in low-melting-point agarose. Image analysis was performed using ImageJ and Zen Blue software (versions 3.6 and 3.11).

### Intersectional labelling with UAS-driven transgenes

To compare the expression pattern of the novel gene trap line with a previously established reporter, we crossed fish from the *tg(gSAIzGFFD2459B)* line to the double transgenic line *tg(rx3:Gal4-VP16); tg(UAS:RFP)*. Embryos were screened at 30 hpf, and those expressing GFP or RFP (GFP + /RFP +) in the expression domains of the transgenic line were selected, fixed, and mounted for subsequent imaging analysis of their expression pattern.

## Results

### *tg(gSAIzGFFD2459B)* drives gene expression in eye and brain cells in zebrafish

**Fig. 1:**
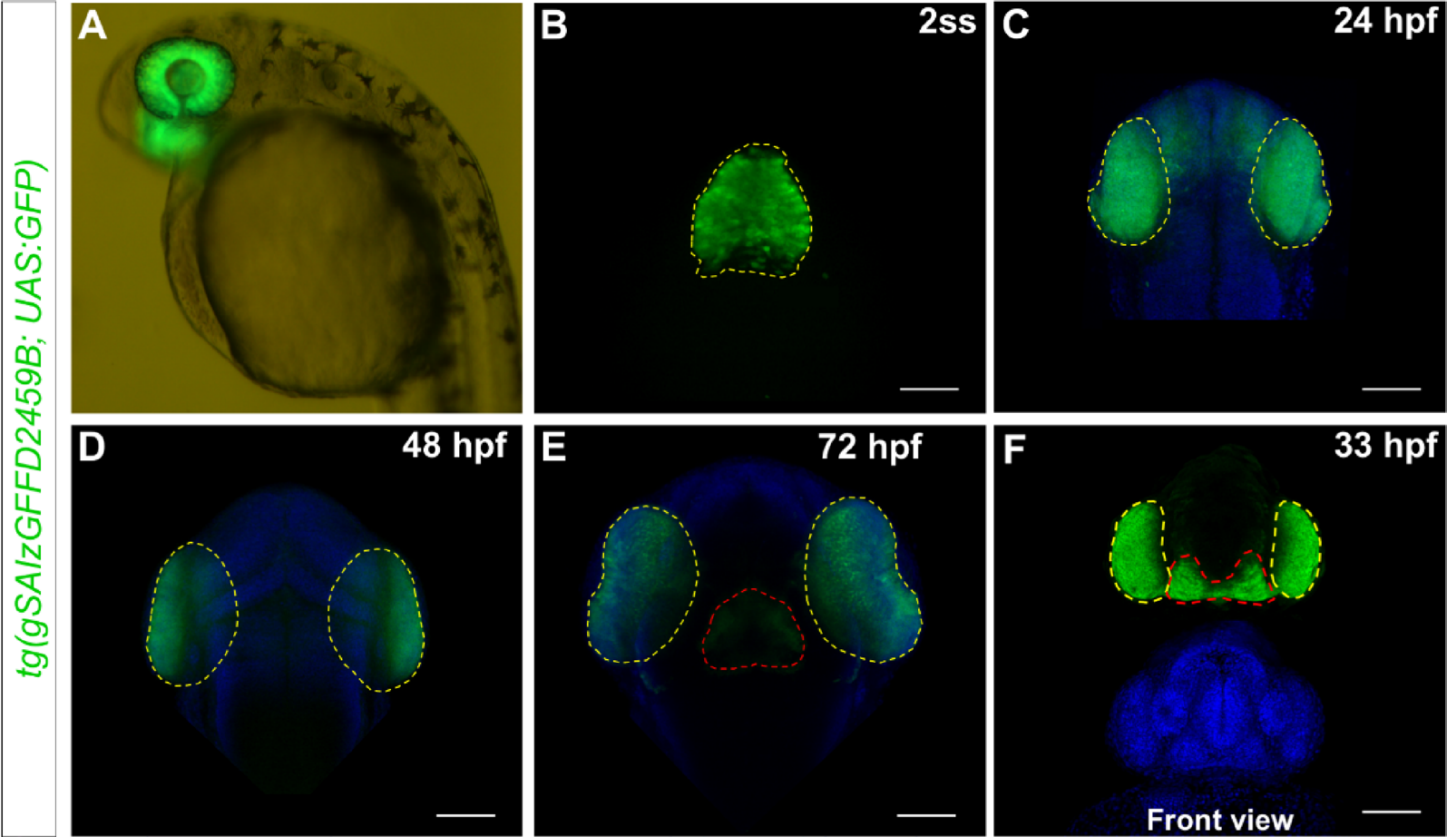
*tg(gSAIzGFFD2459B)-driven* GFP is expressed in embryonic eyes and diencephalon. **A** Lateral overview of GFP expression in the eyes of a 36 hpf embryo. **B** GFP is first visible in the eye field of embryos at the 2-somite stage embryo (dorsal view; anterior is up). **C** Expression becomes restricted to the optic cups at 24 hpf (dorsal view). **D** Expression in the eyes is maintained at 48 hpf, while at 72 hpf (**E**), GFP expression is also observed in the hypothalamus (dorsal view). **F** A front view at 33 hpf shows the expression pattern in the eyes and hypothalamus. Nuclei are stained with DAPI (blue). Yellow dashed lines outline the GFP expression domains in the eye regions, while red dashed lines mark the hypothalamus of the embryo. Scale bar = 100 µm

To identify zebrafish transgenic lines that specifically label eye-fated cells, a targeted genetic screen was conducted for novel Gal4 driver lines. The *tg(gSAIzGFFD2459B)* gene trap line, carrying a *UAS:GFP* reporter, emerged as a promising candidate. When outcrossed to a wild-type background, the *tg(gSAIzGFFD2459B)* insertion robustly drove *UAS:GFP* expression in a distinct and reproducible pattern at 36 hpf (Fig. 1A).

To characterize the expression pattern of the *tg(gSAIzGFFD2459B; UAS:GFP)* line, we examined heterozygous embryos at various developmental stages using confocal microscopy. The earliest GFP expression was detected at 11 hpf (2–3 somite stage) within the eye field, in the anterior neural plate [28–30] (Fig. 1B). As eye field cells underwent evagination and migrated bilaterally to form the optic vesicles and then optic cups, GFP expression was maintained in these structures. By 24 hpf, GFP expression persisted in the retinal progenitor cells of the optic cups (Fig. 1C). By 33 hpf, the reporter was expressed in retinal cells as well as in the hypothalamus (Fig. 1F). The expression pattern remained consistent at 48 hpf (Fig. 1D). By 72 hpf, when most embryonic retinal cells are differentiated and organized into distinct layers, GFP expression was observed throughout the central retina and the hypothalamus (Fig. 1E). Overall, when outcrossed to a *UAS*-reporter line, *tg(gSAIzGFFD2459B)* drives GFP expression in the eye field, optic vesicles, and optic cups, effectively highlighting regions critical for early eye development while also labelling cells in the hypothalamus.

### The *tg(gSAIzGFFD2459B)* is a gene trap insertion in the *rx3* locus

**Fig. 2:**
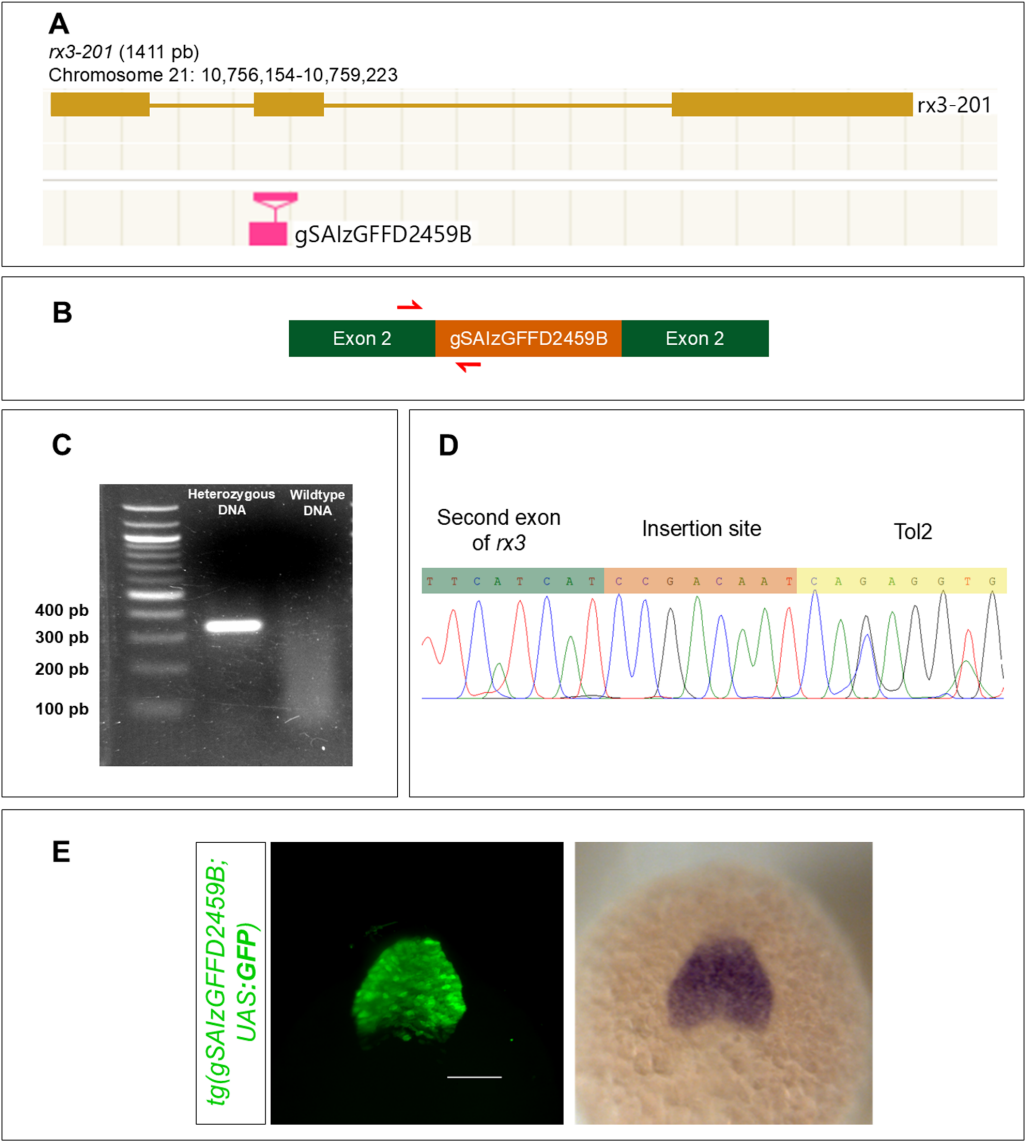
*tg(gSAIzGFFD2459B)* is inserted in the second exon of the *rx3* gene. **A** Screenshot of the zTrap database showing the insertion of the *tg*(*gSAIzGFFD2459B)* transgene in the *rx3* locus. **B** Graphic diagram representing the insertion, specifically in the second exon of *rx3*. Red arrows represent the primers used to validate the insertion. **C** Gel electrophoresis of PCR products amplified from the genomic DNA of *gal4* heterozygous (GFP-positive) embryos shows a 343 bp band (n = 7), whereas no amplification is observed from wild-type embryo genomic DNA. **D** Chromatogram of the sequencing of the PCR product from the insertion validation. The green section corresponds to a part of the *rx3* second exon, the orange section represents the plasmid insertion site of the transgenic line, and the yellow section shows a part of the *Tol2* arm, corresponding to the insertion of the transgene. **E** The expression patterns of the transgene (green, left) and *rx3* (purple, right) in 2-somite stage embryos are similar. The embryos shown in panel E are distinct individuals but are at the same developmental stage. Scale bar = 100 µm

Given the expression pattern observed in the *tg(gSAIzGFFD2459B; UAS:GFP)* transgenic line, we aimed to identify the gene driving this expression. According to the zTrap database, the transgene was inserted in the second exon of the *rx3* gene (Fig. 2A). We experimentally validated this location by performing a genotyping PCR using a primer pair that spanned the *Tol2* sequence of the transgene and the adjacent genomic region of *rx3* (Fig. 2B, C). The amplification and sequencing of a 343-bp product confirmed that the *tg(gSAIzGFFD2459B)* construct is inserted 58-bp downstream of the start of the second exon of *rx3*, thereby creating a gene trap (Fig. 2D). The location of the insertion suggests that *gal4* reporter is expressed under the control of the native *rx3* regulatory sequences. Consequently, when the *tg(gSAIzGFFD2459B)* line is crossed to *tg(UAS:GFP)* responsive line, the resulting GFP expression accurately recapitulates the endogenous *rx3* expression pattern.

To further validate that the observed GFP expression was driven by a construct inserted into the *rx3* genomic locus, we performed in situ hybridization with a specific *rx3* mRNA probe at 11 hpf. This experiment was done at an early stage of zebrafish eye formation [8, 9]. We found a highly similar expression pattern between the endogenous *rx3* gene and the GFP reporter expression in *tg(gSAIzGFFD2459B)* embryos (Fig. 2E). This is consistent with the published expression pattern of *rx3* in the eye field, optic vesicles, and hypothalamus during early zebrafish development [8, 25, 31]. Taken together, these findings suggest that the *tg(gSAIzGFFD2459B)* transgenic line accurately reports *rx3*-positive cells.

### *tg(gSAIzGFFD2459B)* serves as a more faithful reporter of *rx3* expression than existing transgenic lines

**Fig. 3:**
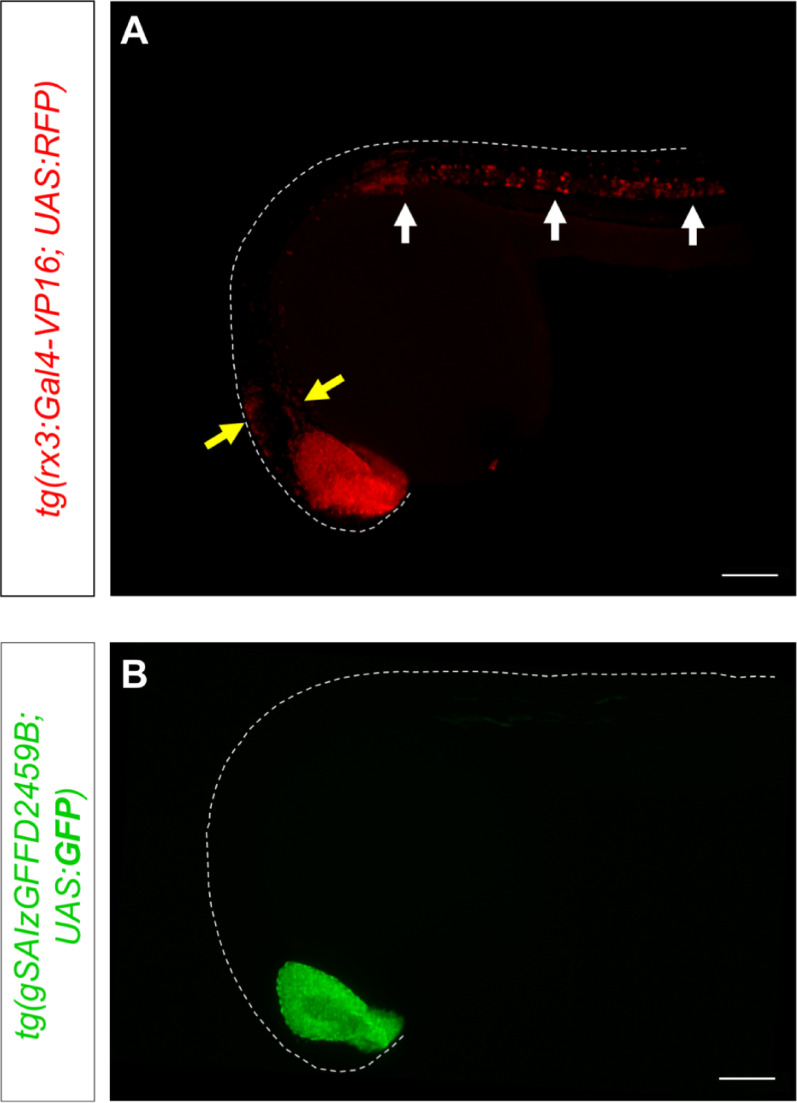
The *tg(gSAIzGFFD2459B)* transgenic line more faithfully recapitulates endogenous *rx3* gene expression. **A** Image of a 26 hpf embryo from the *tg(rx3:Gal4-VP16)* reporter line driving RFP expression in the eyes (red), as well as ectopic expression in the brain (yellow arrows) and the notochord (white arrows) (n = 8). **B** In contrast, a lateral view of a *tg(gSAIzGFFD2459B)* embryo at the same stage shows GFP expression exclusively in the eyes, with no ectopic expression (n = 9). Scale bar = 100 µm

Several transgenic lines have been developed to study the expression of *rx3* in zebrafish. However, all of them are constructed using regulatory sequences from medaka to drive reporter expression [22, 32, 33] To compare expression patterns, we examined the expression of *tg(UAS:RFP)* driven by *tg(rx3:Gal4-VP16)* [22, 34]. At 26 hpf, we observed that the *tg(rx3:Gal4-VP16)* line drives RFP expression in the eyes and anterior brain, but also in ectopic regions in the brain and the notochord, where *rx3* is not normally expressed (Fig. 3A). In contrast, the *tg(gSAIzGFFD2459B)* line directs expression in a much more restricted pattern, closely mirroring the endogenous expression domains of *rx3* in the eye field and optic vesicles but lacking ectopic expression (Fig. 3B). This suggests that the *tg(gSAIzGFFD2459B)* line, with its Gal4 insertion in the *rx3* locus, provides a more faithful recapitulation of the endogenous *rx3* expression domain compared to lines that rely on heterologous regulatory sequences.

### Homozygous *tg(gSAIzGFFD2459B)* insertion results in an eyeless phenotype

**Fig. 4:**
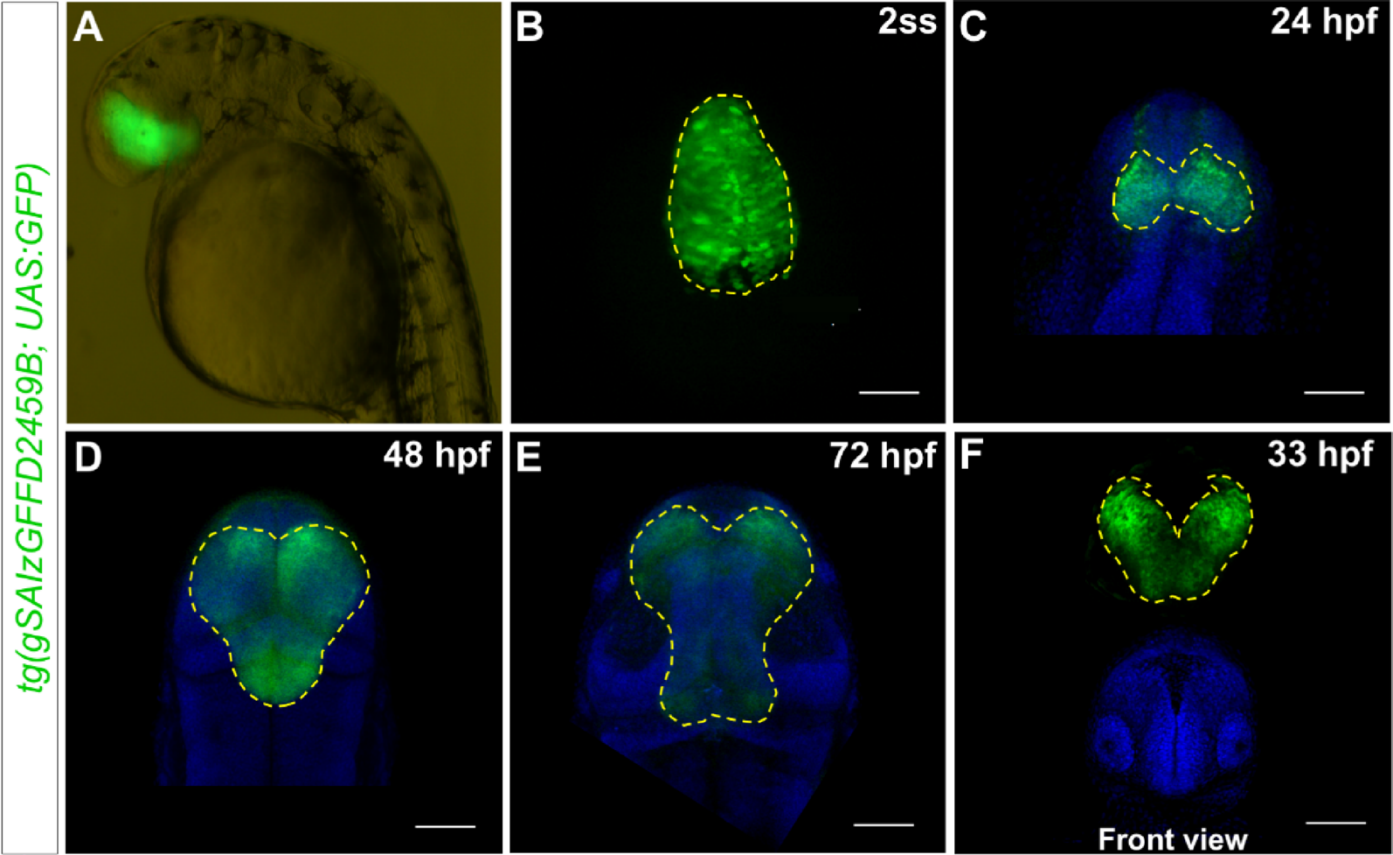
Homozygous *tg(gSAIzGFFD2459B)* embryos display anophthalmia. **A** Lateral overview of the expression of GFP in a 36 hpf eyeless embryo. **B** At the 2-somite stage, GFP-labelled *rx3* + cells are observed in the eye field (dorsal view; anterior is up). **C**–**E** The GFP-labelled cells destined to form the eye structures remain confined in the prosencephalon. GFP expression is maintained in this territory at the 48 hpf (**C**) and 72 hpf (**E**) stages, with no visible eyes or optic structures forming. **F** A front view at 33 hpf shows a cluster of GFP-positive cells retained in the brain. Scale bar = 100 µm

Given the insertion of the *tg(gSAIzGFFD2459B)* transgene within the second *rx3* exon, we investigated the potential for gene disruption in homozygous embryos by incrossing heterozygous *tg(gSAIzGFFD2459B; UAS:GFP)* carriers. Homozygous *tg(gSAIzGFFD2459B)* embryos displayed a failure of optic vesicle evagination that resulted in eyeless embryos (Fig. 4A; Supplementary Fig. 1). This phenotype was accompanied by a progressive alteration in *rx3* expression domains (Fig. 4C–F). While GFP expression was initially similar in both homozygous and heterozygous embryos at 11 hpf (compare Figs. 1B and 4B), a significant deviation was evident by 24 hpf, as the GFP-positive cluster failed to migrate laterally and remained within the forebrain (Fig. 4C). This failure in lateral cell migration ultimately prevented eye formation.

### *tg(gSAIzGFFD2459B)* can be combined with existing UAS transgenic tools

**Fig. 5:**
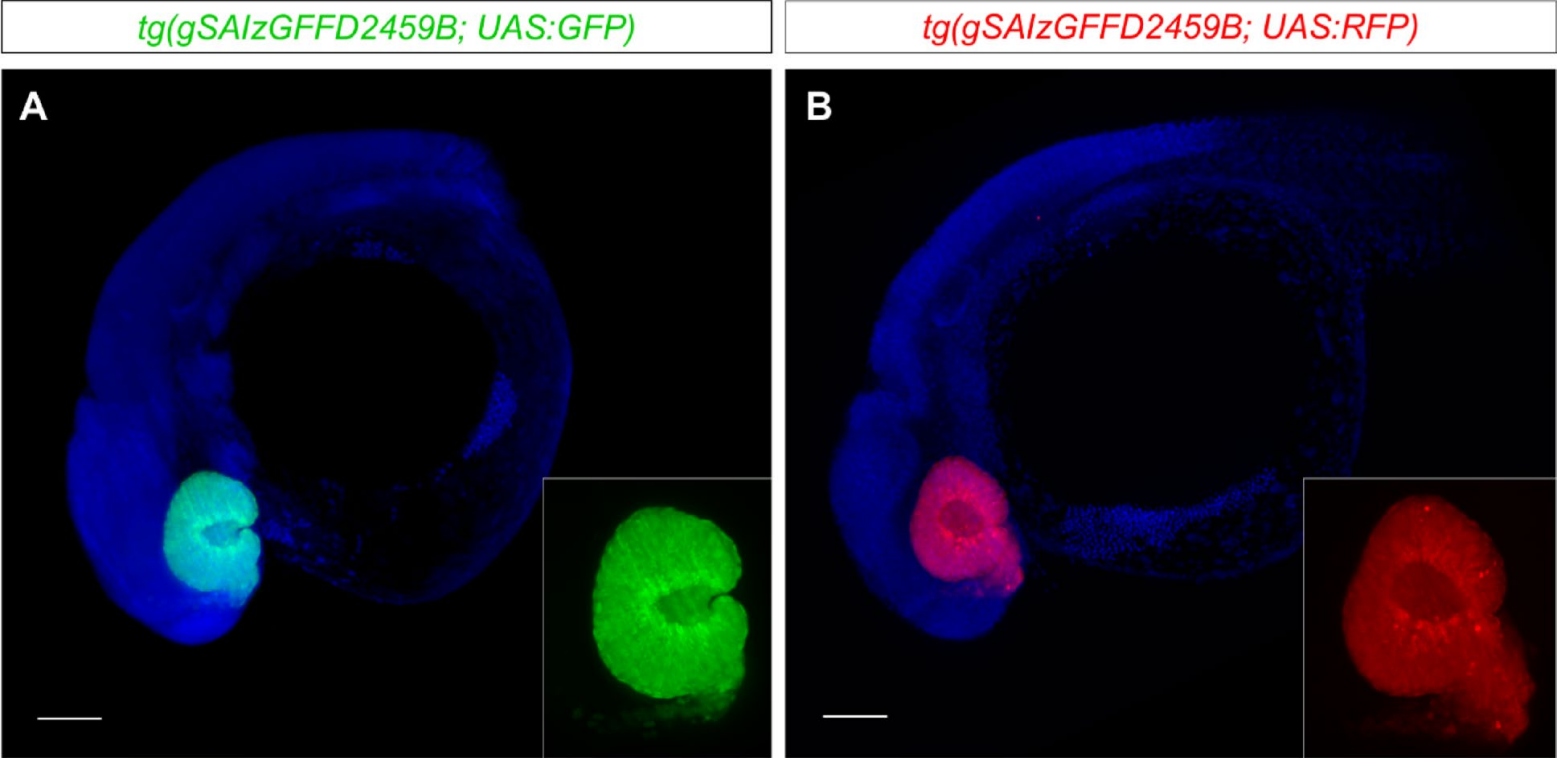
*tg(gSAIzGFFD2459B)* can be combined with other *UAS*-based transgenic reporters. **A*** UAS*-driven GFP expression in a 30 hpf embryo (n = 20). **B*** UAS*-driven RFP expression in a 30 hpf embryo, showing an identical expression pattern in the same territories as observed in **A** (n = 5). Scale bar = 100 µm

The *tg(gSAIzGFFD2459B)* transgenic line can be combined with other *UAS* cassettes, offering experimental flexibility and expanding its utility for diverse research applications. To demonstrate this, we crossed the *tg(gSAIzGFFD2459B)* line with a *tg(UAS:RFP)* reporter and analysed the resulting embryos at 30 hpf. The RFP expression pattern (Fig. 5B) precisely matched the previously observed GFP-labelled domains (Fig. 5A), specifically in the optic cups, with no evidence of ectopic expression. This consistency across a different *UAS* reporter highlights the robustness and reliability of the *tg(gSAIzGFFD2459B)* line. Furthermore, the ability to switch between *UAS* cassettes without altering the spatial or temporal expression profile suggests the line is a tuneable tool, allowing researchers to adapt the reporter system to specific experimental needs. This versatility makes the *tg(gSAIzGFFD2459B)* line a powerful driver line for studying *rx3*-mediated developmental processes and other related gene expression dynamics in zebrafish.

## Discussion

In this study, we isolated and characterized a *gal4*-based *rx3* gene trap transgenic zebrafish line, named *tg(gSAIzGFFD2459B)*, which provides a versatile tool for investigating *rx3* function in eye and brain development. This line uniquely combines the ability to accurately report endogenous *rx3* expression with a simultaneous loss-of-function phenotype in homozygous specimens. The dual functionality and high fidelity of the *tg(gSAIzGFFD2459B)* line make it a reliable resource for the zebrafish community, opening new avenues for studying *rx3*-mediated developmental processes like microphthalmia and transcriptional control.

The *gal4* insertion in *tg(gSAIzGFFD2459B)* is located 58 base pairs downstream the beginning of the second *rx3* exon, and the vector includes splice acceptor and donor sites [23]. This design would allow the transgene to be spliced into the endogenous *rx3* transcript, creating a predicted fusion protein that disrupts *rx3* function in homozygous embryos while accurately reporting its expression in heterozygotes. We confirmed that the GFP expression in *tg(gSAIzGFFD2459B; UAS:GFP)* embryos matches the endogenous *rx3* expression pattern via in situ hybridization, validating that the transgene accurately marks *rx3*-positive cells at early stages of eye formation. This represents a significant improvement over existing *rx3* reporter lines, which all use heterologous regulatory sequences from medaka [10, 22]. These lines can display ectopic expression in regions such as the notochord and other brain areas where *rx3* is not normally expressed (Fig. 3), and their genomic insertion site is unknown. In contrast, the *tg(gSAIzGFFD2459B)* line, with its insertion in the *rx3* locus, leverages the native *rx3* regulatory environment to achieve a more faithful physiologically relevant expression profile, making it a valuable tool. In this context, it is still important to consider that the stability of a fluorescent protein, such as GFP, is longer than that of the endogenous *rx3* mRNA [25, 35]. This difference implies that while the expression patterns match initially, the GFP signal may persist in tissues that do not express *rx3* anymore, which is consistent with the reported decrease of *rx3* expression in the developing eyes after 14 h post-fertilization [25].

The *tg(gSAIzGFFD2459B)* line opens several possibilities for the zebrafish community, particularly for developmental biologists, by enabling the direct visualization and manipulation of *rx3*-positive cells. While general *Gal4/UAS* systems carry the risk of positional effects and variegation (transcriptional silencing), our line exhibits minimal to no variegation. This stability and specificity are accompanied by a high signal-to-noise ratio, making it ideal for high-resolution imaging of the eye field, optic vesicles, and hypothalamus. This is consistent with the fact that both the eyes and the hypothalamus originate from the forebrain, sharing a common developmental origin [36–38]. Furthermore, the modular nature of the *Gal4/UAS* system provides a high degree of flexibility. When the *tg(gSAIzGFFD2459B)* line was crossed with a *tg(UAS:RFP)* reporter, it showed the same precise expression pattern as with the *tg(UAS:GFP)* reporter, without ectopic expression (Fig. 5). This tuneability allows researchers to combine the line with various *UAS* cassettes to meet specific experimental needs. For instance, it could be used with *tg(UAS:kaede)* for cell lineage tracing or with *tg(UAS:NTR)* for targeted cell ablation [39–41]. This versatility makes the *tg(gSAIzGFFD2459B)* line a valuable resource for investigating the role of *rx3* in eye development, neurogenesis, and brain organization [9].

Another key advantage of the *tg(gSAIzGFFD2459B)* line is its utility as a loss-of-function model. Homozygous embryos display a complete failure of optic vesicle evagination, resulting in an eyeless phenotype that is highly consistent with the established essential role of *rx3* in optic vesicle formation and defining eye versus brain fate in zebrafish and medaka [8, 10]; the relevance of this model is underscored by the fact that mutations in the mouse and human ortholog *RX* genes are known to be associated with anophthalmia [15]. Remarkably, the severe, fully penetrant anophthalmia in *tg(gSAIzGFFD2459B)* homozygotes provides an important contrast to existing *rx3* zebrafish mutant alleles: in those alleles, mutations introduce stop codons within or after the homeodomain, often resulting in residual function and the formation of rudimentary eye structures [8, 42]. Our *gal4* transgenic insertion contains splicing acceptor/donor sequences and is located before the critical homeodomain coding region, suggesting that any resulting truncated protein would be non-functional. This mechanism coupled with the robust *gal4*-driven reporter expression which argues against nonsense-mediated decay, establishes our line as a true null allele. Furthermore, its consistent severity in the AB genetic background emphasizes the robustness of the functional disruption, as other mutations affecting eye formation have been shown to be less severe or sensitive to the AB genetic background [43].

While other *rx3* mutant alleles have been used for bulk transcriptomics studies [44], *tg(gSAIzGFFD2459B)* offers unique advantages that expand the scope of research. For instance, its high-fidelity reporter system enables single-cell analysis, such as single cell RNA-seq, by allowing the isolation of *rx3*-positive cells for high-resolution transcriptomic characterization. Therefore, the *tg(gSAIzGFFD2459B)* line offers potential as a platform to investigate the molecular mechanisms underlying microphthalmia.

## Conclusions

Our findings establish the *tg(gSAIzGFFD2459B)* transgenic line as a versatile tool for studying eye development in zebrafish. This line has a unique dual functionality, acting as both a precise reporter and a loss-of-function model. Its endogenous insertion in the *rx3* locus provides a more faithful recapitulation of endogenous gene expression, avoiding ectopic expression. This makes it a powerful and high-fidelity tool for studying gene expression, while its eyeless phenotype serves as a valuable model for investigating the causes and consequences of anophthalmia. The modularity of the *Gal4/UAS* system further enhances its utility, making it a versatile resource for a wide range of experimental applications, from cell lineage tracing to targeted cell ablation.

## Electronic supplementary material

Below is the link to the electronic supplementary material.

Supplementary file 1: Figure 1: Phenotype of heterozygous and homozygous *tg(gSAIzGFFD2459B)* embryos. Bright-field images illustrating the complete absence of eye structures in homozygous *tg(gSAIzGFFD2459B)* embryos compared to their heterozygous siblings at 48- and 72-hours hpf. (A) Dorsal view of a 48 hpf heterozygous embryo showing normal eye development. (B) Dorsal view of a 48 hpf homozygous embryo exhibiting the eyeless phenotype. (C) Dorsal view of a 72 hpf heterozygous embryo. (D) Dorsal view of a 72 hpf homozygous embryo lacking eye structures. (E) Lateral view of a 72 hpf heterozygous embryo. (F) Lateral view of a 72 hpf eyeless homozygous embryo. Sample sizes at 48 hpf: wild type/heterozygous n=23, homozygous n=14. Sample sizes at 72 hpf: wild type/heterozygous n=26, homozygous n=32. Scale bar = 500 µm.

## Data Availability

The datasets during and/or analysed during the current study are available from the corresponding author on reasonable request.

## Abbreviations

*rx3*: Retinal homeobox gene 3
*gal4*: Galactose-responsive transcription factor 4
*UAS*: Upstream activating sequence
*Tol2*: Transposable element of *Oryzias latipes*, number 2
GFP: Green fluorescent protein
RFP: Red fluorescent protein
PFA: Paraformaldehyde
PBSTr: Phosphate-buffered saline with Triton X-100
VP16: Viral protein 16
PCR: Polymerase chain reaction
Hpf: Hours post fertilization
NTR: Nitroreductase
