# Supplementary figures and images for: A gal4 insertion in the rx3 locus as a tool for visualization and manipulation of eye fated cells in zebrafish
Source: Biol Res. 2025 Nov 23;58:74. doi: 10.1186/s40659-025-00656-9 (PMC12701590; doi:10.1186/s40659-025-00656-9)

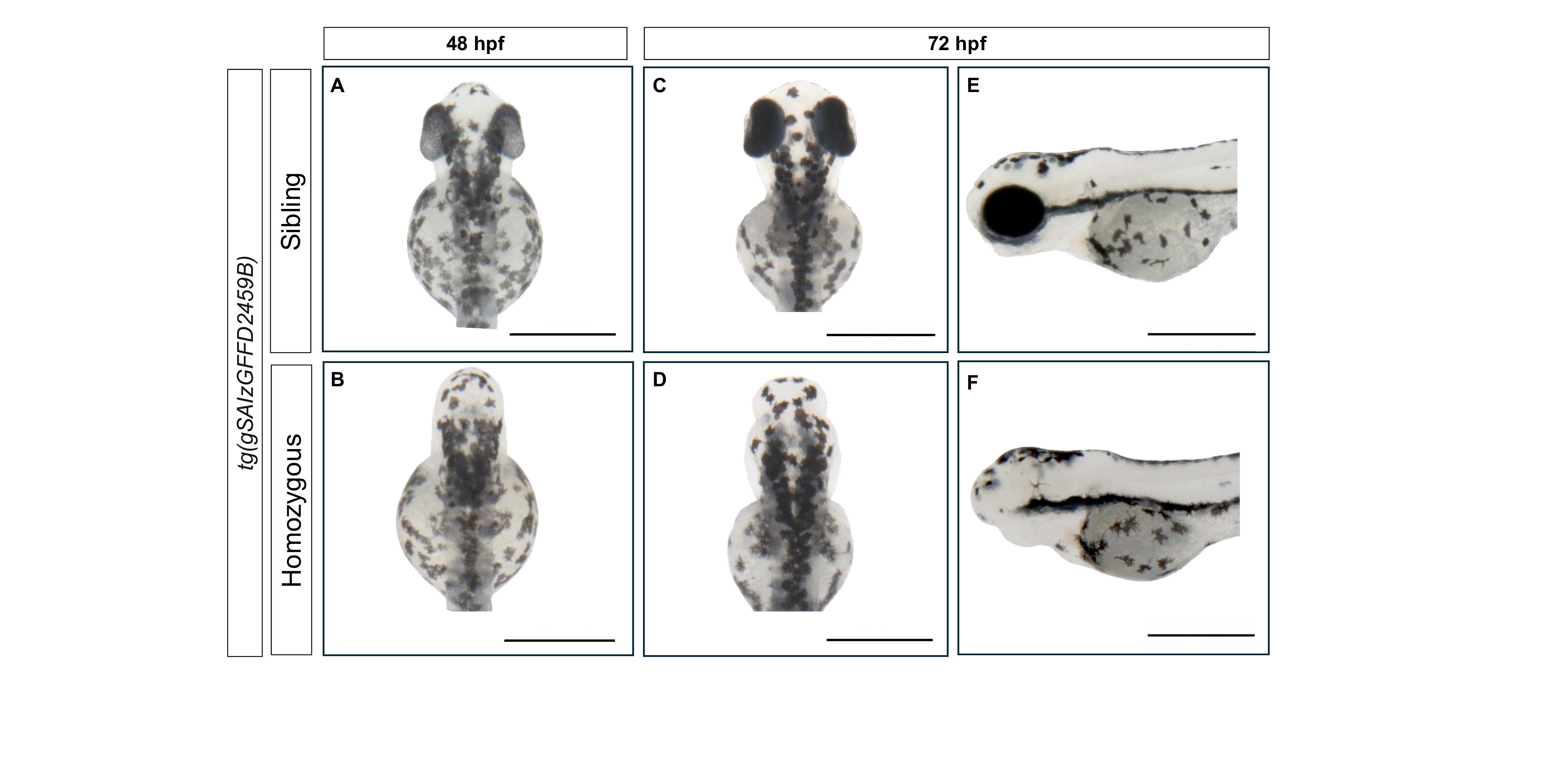

Supplement: Supplementary file 1 — Figure 1: Phenotype of heterozygous and homozygous tg(gSAIzGFFD2459B) embryos. Bright-field images illustrating the complete absence of eye structures in homozygous tg(gSAIzGFFD2459B) embryos compared to their heterozygous siblings at 48- and 72-hours hpf. (A) Dorsal view of a 48 hpf heterozygous embryo showing normal eye development. (B) Dorsal view of a 48 hpf homozygous embryo exhibiting the eyeless phenotype. (C) Dorsal view of a 72 hpf heterozygous embryo. (D) Dorsal view of a 72 hpf homozygous embryo lacking eye structures. (E) Lateral view of a 72 hpf heterozygous embryo. (F) Lateral view of a 72 hpf eyeless homozygous embryo. Sample sizes at 48 hpf: wild type/heterozygous n=23, homozygous n=14. Sample sizes at 72 hpf: wild type/heterozygous n=26, homozygous n=32. Scale bar = 500 µm. [file 40659_2025_656_MOESM1_ESM.tif]
